# Cross-cultural adaptation, reliability and validity of the Spanish version of the Quality of Life in Adult Cancer Survivors (QLACS) questionnaire: application in a sample of short-term survivors

**DOI:** 10.1186/s12955-015-0378-2

**Published:** 2015-11-16

**Authors:** Antonio Escobar, Maria del Mar Trujillo-Martín, Antonio Rueda, Elisabeth Pérez-Ruiz, Nancy E. Avis, Amaia Bilbao

**Affiliations:** Unidad de Investigación, Hospital Universitario Basurto, Red de Investigación en Servicios de Salud en Enfermedades Crónicas (REDISSEC), Avenida Montevideo, 18, 48013 Bilbao, Bizkaia Spain; Servicio de Evaluación y Planificación. Dirección del Servicio Canario de la Salud. Fundación Canaria de Investigación Sanitaria (FUNCANIS), Red de Investigación en Servicios de Salud en Enfermedades Crónicas (REDISSEC), Camino Candelaria, 44. C.S. San Isidro-El Chorrillo, 38109 El Rosario, Tenerife Spain; Área de Oncología, Hospital Costa del Sol, Red de Investigación en Servicios de Salud en Enfermedades Crónicas (REDISSEC), Autovía A-7, Km 187, 29603 Marbella, Málaga Spain; Servicio de Oncología Médica, Hospital Costa del Sol, Autovía A-7, Km 187, 29603 Marbella, Málaga Spain; Division of Public Health Sciences, Department of Social Sciences and Health Policy, Medical Center Boulevard Wake Forest School of Medicine, Winston-Salem, NC 27157-1063 USA

**Keywords:** Cancer survivors, QLACS, Psychometric, Rasch measurement, Quality of life

## Abstract

**Background:**

The aim of this study was to validate the Quality of Life in Adult Cancer Survivors (QLACS) in short-term Spanish cancer survivor’s patients.

**Methods:**

Patients with breast, colorectal or prostate cancer that had finished their initial cancer treatment 3 years before the beginning of this study completed QLACS, WHOQOL, Short Form-36, Hospital Anxiety and Depression Scale, EORTC-QLQ-BR23 and EQ-5D. Cultural adaptation was made based on established guidelines. Reliability was evaluated using internal consistency and test-retest. Convergent validity was studied by mean of Pearson’s correlation coefficient. Structural validity was determined by a second-order confirmatory factor analysis (CFA) and Rasch analysis was used to assess the unidimensionality of the Generic and Cancer-specific scales.

**Results:**

Cronbach’s alpha were above 0.7 in all domains and summary scales. Test-retest coefficients were 0.88 for Generic and 0.82 for Cancer-specific summary scales.

QLACS generic summary scale was correlated with other generic criterion measures, SF-36 MCS (r = − 0.74) and EQ-VAS (r = − 0.63). QLACS cancer-specific scale had lower values with the same constructs.

CFA provided satisfactory fit indices in all cases. The RMSEA value was 0.061 and CFI and TLI values were 0.929 and 0.925, respectively. All factor loadings were higher than 0.40 and statistically significant (*P* < 0.001). Generic summary scale had eight misfitting items. In the remaining 20 items, the unidimensionality was supported. Cancer Specific summary scale showed four misfitting items, the remaining showed unidimensionality.

**Conclusions:**

The findings support the validity and reliability of QLACS questionnaire to be used in short-term cancer survivors.

**Electronic supplementary material:**

The online version of this article (doi:10.1186/s12955-015-0378-2) contains supplementary material, which is available to authorized users.

## Background

Prevalence of patients living with cancer diagnosis is growing all around the world. The main aim of treatments has usually been to improve the survival of these patients, but the great success in this objective as well as with screening tests implies that there are a growing number of patients surviving this diagnosis and its treatment. Therefore, our concern should be focused on this new phase: From the end of treatment to five years in the future, the study of short-term cancer survivors. At this stage, adverse events have diminished or even disappeared and we should confront new aspects of their care, such as adaptation to the new situation [1] or complications that may arise later.

The impact of treatment on health related quality of life (HRQoL) for cancer patients is well known. The questionnaires used in this first phases of the disease such as Functional Assessment of Cancer Therapy (FACIT) [2] or European Organization for Research and Treatment of Cancer (EORTC) [3] are not specifically directed to evaluate the new symptoms and worries that arise after finishing their treatments. Nowadays, a concern that arises is the measurement of patient reported outcomes (PROs), which could offer an enormous potential to evaluate, monitor and possibly to improve the quality and success of care with these short-term cancer survivors, with new and sound questionnaires.

In a literature review [4], six instruments devoted to cancer survivors have been identified. Among them, the Quality of Life in Adult Cancer Survivors (QLACS) [5] was found to be the best questionnaire, for its suitable psychometric properties. The QLACS is a multidimensional questionnaire that has five Cancer-specific domains, which were suggested by patients as relevant to their lives, along with seven Generic domains that are relevant to patients with cancer but not limited to them. It also considers Generic and Cancer-specific summary scores.

The QLACS questionnaire was originally designed for use with long-term survivors, but it is now being used in shorter-term survivors in several studies [6, 7] and has shown good psychometric properties in 15 months post-diagnosis cancer survivors [8]. The authors concluded that although QLACS was designed for patients over 5 years post-diagnosis, it is also valid for shorter-term survivors. Recently, a new study [9] on the reliability and validity of QLACS in short-term breast cancer survivors was published. Authors concluded that it is an adequate instrument in these patients at 18–24 months post-diagnosis.

In the light of the reliability and validity of the QLACS questionnaire for short-term cancer survivors, the aim of our study was to perform a cross-cultural adaptation of the QLACS questionnaire for use in Spain and to validate the Spanish version in terms of its reliability and validity properties, in a sample of short-term breast, colorectal or prostate cancer survivors.

## Methods

This is a retrospective cohort study. Adult patients with breast, colorectal or prostate cancer and who had finished their initial cancer treatment 3 years before the beginning of this study were included. This study took place in 12 hospitals belonging to the National Health Service; 3 in Andalusia, 4 in the Canary Islands and 5 in the Basque Country (Spain). All patients received a letter informing them about the study and requesting their voluntary participation, all of them signed the informed consent. All patients who accepted to take part in the study were included, given the fact that the inclusion criteria was to answer the first questionnaire. The Institutional Review Boards of the Hospitals approved the study. All the questionnaires were sent to patients via mail in a range of three-four years after finishing their initial cancer treatment. Apart from QLACS, the Spanish validated questionnaires included in this study were, WHOQOL [10], Short Form-36 [11], Hospital Anxiety and Depression Scale (HADS) [12], EORTC-QLQ-BR23 [13] and EQ-5D-3 L [14].

### Questionnaires

The QLACS questionnaire comprises 47 items measuring 12 domains. There are 7 Generic domains, all of them with 4 items, negative feelings, positive feelings, cognitive problems, sexual problems, pain, fatigue and social avoidance. There are also 4 Cancer-specific, appearance concerns (4 items), financial problems (4 items), distress over recurrence (4 items) and family-related distress (3 items). There is another domain with 4 items, benefits of cancer. Each item is scored on a seven-point frequency scale (1 = never, 2 = seldom, 3 = sometimes, 4 = about as often as not, 5 = frequently, 6 = very often and 7 = always), regarding the past four weeks.

Domain scores range from 4 to 28, with higher scores indicating lower HRQoL (the 3 items domain of family-related distress score is multiplied by 1.33). The score for positive feelings is reversed as well as item 1 in fatigue domain. The Generic summary score ranges from 28 to 196 and the Cancer-specific summary score from 16 to 112.

WHOQOL. The WHOQOL-100 assesses individuals’ perceptions of their position in life in the context of the culture and value systems in which they live and in relation to their goals, expectations, standards and concerns. It is a 100-question instrument that yields a multi-dimensional profile of scores across 6 domains and 24 quality of life sub-domains. In this study we have only used some sub-domains of the questionnaire

SF-36 is a 36 item instrument. It provides scores on 8 dimensions and 2 summary scores: the physical component summary (PCS) and the mental component summary (MCS). Scores range from 0 to 100 with a higher score indicating better health status.

HADS is a questionnaire divided into two subscales with 7 questions pertaining to symptoms regarding anxiety and 7 to symptoms associated with depression. Each of the 14 items consists of a 4-point Likert scale (ranging from 0 to 3) that applies to the previous week. Higher scores indicate worse status.

QLQ-BR23 is the breast module of the EORTC questionnaire. It is composed of 23 items divided into 4 functional scales and 4 symptoms scales.

EQ-5D-3 L has two sections. The first part, a descriptive system, consists of five questions covering the dimensions of mobility, self-care, usual activities, pain/discomfort, and anxiety/depression. The second part consists of a 20 cm vertical visual analogue scale (VAS) ranging from 0 (worse) to 100 (best health). In this study we have used VAS scale.

The dimensions used in the validation process can be seen in Table 4.

### Adaptation of the QLACS

Firstly, we obtained permission from the original authors [5] to translate and validate the questionnaire. Translation and cultural adaptation were made, based on established guidelines for cross-cultural adaptation [15]. The original English questionnaire was independently translated into Spanish by two translators (one oncologist and one professional translator) whose native language was Spanish and who were highly fluent in English. Both highlighted the difficulty of finding Spanish expressions that were conceptually equivalent to the original expressions. The two translations were compared and discussed in a meeting that included the research team and the translators until a consensus was reached on a single adapted version (version 1.0). To evaluate the equivalence of Spanish version 1.0 to the original, it was independently back-translated to English by two native professional translators who were highly fluent in Spanish. The two back translations were compared with the original English version and a consensus was reached on any necessary modifications in the Spanish version 1.0. Finally, the revised version of the Spanish QLACS was tested on 10 patients in order to evaluate how well patients understood the items, as well as to determine the acceptability and feasibility of the questionnaire. These patients were not in the final study sample.

### Statistical analysis

The distribution of scores in the Spanish version of the QLACS was evaluated by analyzing the mean, standard deviation, proportion of patients with one or more lost items, observed range, and the ceiling and floor effects taking into account the accepted values of > 15 % [16].

### Reliability

Reliability was analyzed in two ways, internal consistency by means of Cronbach’s alpha, and item- total (domain and summary scale) correlations, with values ≥ 0.7 [16] and ≥ 0.3 respectively, indicating acceptable values. Reproducibility was analyzed by means of test–retest. Patients were explicitly asked whether they had experienced any change in their health status since completing the previous questionnaire ten days before. When no change was detected, we calculated the intraclass correlation coefficients (ICC) for absolute agreement by two-way random effects model. Values higher than 0.5 are considered acceptable [17].

### Validity

Convergent and discriminant validity was studied by means of Pearson’s correlation coefficient between some QLACS domains and other validated questionnaires. We have been unable to study convergent validity in cognitive problems, financial problems, family-related distress and benefits of cancer due to the lack of adequate dimensions or scales in our study. We have used subscales of well-known Spanish validated questionnaires such as WHOQOL [10], Short Form 36 [11], HADS [12], EORTC-QLQ-BR23 [13] and EQ-5D [14] as criterion measures.

We hypothesized that QLACS generic summary score should have negative high correlations with other generic measures such as SF-36 Mental Component Summary and Physical Component Summary (MCS and PCS) or EQ-5D Visual Analogic Scale (VAS). The cancer-specific summary scale should have lower correlations with the same generic measures. On the other hand, QLACS’s domains should have high correlations with similar domains of the other questionnaires included (e.g., QLACS pain with SF-36 bodily pain, appearance concerns with WHOQOL body image and so on). We considered convergent validity as moderate when 0.3 > r < 0.49 and high if r ≥ 0.50 [18].

Construct validity. To study the structural validity of the questionnaire, two different approaches were used. First, a second-order confirmatory factor analysis (CFA) for categorical data was used to confirm the internal structure, consisting of 12 first-order factors and two second-order factors. The first-order factors are negative feelings (NF), positive feelings (PF), cognitive problems (CP), sexual problems (SP), pain (PN), fatigue (FG), social avoidance (SA), appearance concerns (AC), financial problems (FP), distress over recurrence (DOR), family-related distress (FRD), and benefits of cancer (BOC). Then, a second-order Generic factor would affect the first-order NF, PF, CP, SP, PN, FG and SA factors, and the other second-order cancer-specific factor would affect AC, FP, DOR and FRD first-order factors [5]. The robust weighted least squares estimator was used, and several fit indices were calculated: the root mean square error of approximation (RMSEA), for which a value <0.08 was considered acceptable; and the Tucker-Lewis Index (TLI) and Comparative Fit Index (CFI), both of which had to be >0.90 to be satisfactory. We also examined factor loadings, and those ≥0.40 were considered acceptable [19–21].

Second, we used Rasch analysis with the polytomous Partial Credit Model because the response scales of the questionnaire are ordinal with seven response options [22, 23]. We applied the Rasch method to the Generic factor, the Cancer-specific factor, and the BOC factor separately, to ensure that the scales were unidimensional [24] as this is a fundamental requirement for construct validity. Unidimensionality was assessed with two indices of fit, namely the mean square information-weighted statistic (infit) and the outlier-sensitive statistic (outfit), with values between 0.7 and 1.3 indicating a good fit [25], and a principal component analysis (PCA) of the residuals. Unidimensionality was considered violated if, besides the first factor, other factors had eigenvalues >3 [26]. We evaluated the ability of the QLACS to define a distinct hierarchy of items along the measured dimension by means of an item separation index [24]. A value >2.0 is comparable to a reliability of 0.80 and considered acceptable. To detect the presence of differential item functioning (DIF), which occurs when different groups within the sample respond in a different manner to an individual item [22], we compared different levels of the trait by type of cancer and gender and age (<65 vs. ≥ 65 years). A Welch’s *t* statistically significant at *P* < 0.05, and a difference in difficulty ≥0.5 logit were considered to be noticeable DIF [24]. Residual correlations between items within a scale were examined for local dependency. Correlations >0.5 between item residuals can indicate that responses to one item may be determined by others [27]. The functioning of rating scale categories was also examined for each item. A clearly progressive level of difficulty across the item categories was considered adequate [24]. Where the response format was disordered, such that higher response options did not uniformly reflect increases in the underlying construct, this was resolved by collapsing adjacent response categories.

All effects were considered statistically significant at *P* < 0.05. The statistical analyses were performed with SAS for Windows (version 9.2; SAS Institute, Cary, NC), Mplus (version 6.1; Muthén et al., 1998–2010), and Winsteps (version 3.69.1.4; John M. Linacre, Chicago).

## Results

In the translation-back-translation process, we had to look for advice from the original authors in two points. Firstly, there were some problems with conceptual meaning of some expressions such as “felt fatigued”, “were bothered by mood swings” or “felt anxious”. Second, there was a problem with the labeling of the answer scale. The intermediate point “about as often as not” was difficult to adapt. All of them were resolved with the original authors and incorporated in the final version (see Additional file 1).

707 patients were included in the field study. The main characteristics of the sample are summarized in Table 1. The mean (SD) age of the prostate cancer patients was 70.3 (7.1) with a range from 47 to 87 years. Breast cancer patients had a mean age (SD) of 60.8 (11.6) with a range of 30–91. Finally, colorectal cancer patients had a mean age (SD) of 68.7 (9.5) and a range of 30–91 years. The percentage of women in colorectal cancer patients was of 35.2 %. The mean scores with standard deviation of each QLACS domain and summary scale are displayed in Table 2. There were 4 domains with floor effect (pain, social avoidance, appearance concerns and financial problems) and one with ceiling effect (family-related distress); more than 15 % of patients scoring in minimum or maximum score respectively. Summary scales were free of both effects.

**Table 1:** Sample characteristics (*N* = 707)

Variable	N (%)
Cancer type	
Prostate	160 (22.6 %)
Breast	354 (50.1 %)
Colorectal	193 (27.3 %)
Gender (female)	422 (59.7 %)
Age	
mean (standard deviation)	65.1 (11.0)
range	30–91
Education	
< High school	467 (66.1 %)
High school	130 (18.4 %)
University	76 (10.7 %)
Non-response	34 (4.8 %)
Relationship status	
Married/couple	502 (71.0 %)
Single	36 (5.1 %)
Widowed	86 (12.2 %)
Divorced/separated	48 (6.7 %)
Non-response	35 (5.0 %)
Employment status	
Retired	347 (49.1 %)
Unemployed	51 (7.2 %)
Employed	256 (36.2 %)
Non-response	53 (7.5 %)

**Table 2:** Descriptive data and reliability analysis for QLACS domains and summary scales

Dimensions	N	Mean (SD)	Score range	Floor effect (%)	Ceiling effect (%)	Cronbach’s α	ICC (95 % CI)
							*n* = 137
Negative feelings	692	12.0 (4.8)	4–28	4.7	0.1	0.75	0.77 (069–0.83)
Positive feelings	679	19.9 (5.7)	4–28	0.9	10.2	0.73	0.58 (0.45–0.68)
Cognitive problems	697	10.5 (5.0)	4–28	10.5	0.0	0.78	0.53 (0.40–0.64)
Sexual problems	654	12.7 (6.2)	4–28	9.0	2.3	0.75	0.73 (0.64–0.80)
Pain	689	10.6 (5.9)	4–28	16.7	0.7	0.86	0.76 (0.67–0.82)
Fatigue	694	11.9 (5.3)	4–28	7.6	0.3	0.74	0.76 (0.68–0.82)
Social avoidance	688	9.3 (5.2)	4–28	23.4	0.3	0.82	0.79 (0.71–0.84)
Generic Summary Scale	630	79.0 (28.0)	28–176	0.6	0.2	0.85	0.88 (0.83–0.91)
Appearance concerns	686	9.9 (6.2)	4–28	27.8	0.9	0.77	0.76 (0.68–0.82)
Financial problems	694	6.1 (4.2)	4–28	66.1	0.1	0.75	0.65 (0.53–0.73)
Distress over recurrence	692	17.0 (7.2)	4–28	4.8	8.5	0.81	0.72 (0.63-0.80)
Family-related distress	693	19.3 (7.8)	4–28	5.9	26.4	0.84	0.66 (0.55–0.74)
Cancer specific Summary Scale	678	52.5 (18.1)	16–106	1.3	0.1	0.85	0.82 (0.76–0.87)
Benefits of cancer	692	18.0 (6.6)	4–28	5.2	6.8	0.81	0.67 (0.56–0.76)

*SD* standard deviation

*ICC* intraclass correlation coefficient

*CI* confidence interval

### Reliability

The Cronbach’s alpha values are shown in Table 2 and they were above 0.7 in all domains and both summary scales.

As can be seen in Table 3, in QLACS Generic domains, all corrected item-domain and item-summary scale correlations were above 0.30 with higher values in item-domain correlation (range: 0.61–0.86) than in the item-summary scale correlations (range: 0.38–0.73). The percentage of missing data was high in the sexual domain (from 5.9 to 10.5 %). One item in the social avoidance (7.9 %), positive feelings (14.9 %) and pain (7.2 %) domains showed values higher than 5 % of missing data as well.

**Table 3:** Missing data, item-domain and item summary scale correlations for QLACS domains

Generic Domains	Item	Missing data (%)	Item-domain correlation	Item-summary scale correlation
Negative feelings	07	3.4	0.71	0.62
	09	3.7	0.70	0.44
	19	2.8	0.75	0.69
	24	2.8	0.76	0.65
Positive feelings	06	5.0	0.73	0.56
	08	3.4	0.75	0.60
	22	4.0	0.68	0.40
	28	14.9	0.61	0.38
Cognitive problems	02	2.0	0.80	0.54
	03	3.0	0.82	0.58
	04	2.1	0.79	0.51
	23	2.5	0.66	0.51
Sexual problems	10	6.8	0.70	0.40
	12	7.1	0.75	0.41
	16	5.9	0.75	0.50
	26	10.5	0.71	0.53
Pain	13	2.3	0.86	0.67
	17	2.8	0.85	0.73
	21	4.0	0.82	0.62
	27	7.2	0.78	0.64
Fatigue	01	2.3	0.74	0.54
	05	3.3	0.77	0.62
	11	1.7	0.85	0.70
	14	2.4	0.85	0.70
Social Avoidance	15	7.9	0.68	0.45
	18	3.0	0.82	0.67
	20	2.5	0.76	0.61
	25	2.4	0.78	0.58
Specific Domains				
Appearance concern	33	1.8	0.83	0.59
	35	4.0	0.78	0.51
	38	6.5	0.78	0.51
	44	2.4	0.66	0.50
Financial problems	30	2.8	0.72	0.31
	37	3.5	0.39	0.12
	43	2.3	0.91	0.45
	45	2.1	0.89	0.45
Distress over Recurrence	36	2.0	0.79	0.63
	39	3.8	0.78	0.66
	46	2.4	0.81	0.68
	47	2.8	0.75	0.63
Family-related distress	31	1.8	0.89	0.64
	34	2.5	0.88	0.69
	42	2.4	0.82	0.64
Benefits of cancer	29	1.4	0.79	NA
	32	2.1	0.82	NA
	40	2.1	0.85	NA
	41	3.5	0.70	NA

Regarding Specific domains (Table 3), all item correlations were also above 0.30 but one in financial problems domain (r = 0.12). As expected, higher values were observed in item-domain correlation, ranging from 0.39 to 0.91 than in the item-summary scales correlations (range: 0.12 to 0.69). There were a low percentage of missing data in these specific domains, all below 4.0 % but one in appearance concern domain (6.5 %).

Considering test-retest reliability (Table 2), the study was carried out with 137 stable patients and coefficients ranged from 0.53 to 0.79 in the different domains. The Generic summary scale had a ICC of 0.88 and the Cancer-specific summary scale of 0.82.

### Convergent validity

Table 4 shows results on the convergent validity analysis. Considering the absolute size of the correlation coefficients, and ignoring the direction of correlation, as we hypothesized, QLACS generic summary scale was negatively correlated with other generic quality of life criterion measures, SF-36 MCS (r = − 0.74) and EQ-VAS (r = − 0.63) and this coefficient was lower with SF-36 PCS (r = − 0.57). On the other hand, QLACS cancer-specific scale had lower values with the same constructs, ranging from 0.20 to 0.40.

**Table 4:** Correlation between some QLACS domains and different criterion measures

QLACS Dimensions	Correlation	Criterion measures
Negative feelings	−0.73	SF-36 (Mental Health)
Positive feelings	0.62	HADS (Depression)
Sexual problems	−0.60	WHOQOL(Sex functioning)
Pain	−0.69	WHOQOL (Pain)
	−0.69	SF-36 (Bodily Pain)
Fatigue	0.78	WHOQOL (Energy/fatigue)
	−0.77	SF-36 (Vitality)
Social avoidance	0.60	SF-36 (Social Functioning)
Generic Summary Scale	−0.74	SF-36 (Mental Component Summary)
	-0.57	SF-36 (Physical Component Summary)
	−0.63	EQ-VAS
Appearance concerns	−0.53	WHOQOL(Body image)
Distress over recurrence	0.60	EORTC-QLQ-BR23(Future Perspective)
Cancer-specific Summary Scale	−0.40	SF-36 (Mental Component Summary)
	−0.28	SF-36 (Physical Component Summary)
	−0.20	EQ-VAS

SF-36: Short Form 36

*HADS* hospital anxiety and depression scale

*WHOQOL* World Health Organization quality of life

*EQ*-*VAS* EuroQoL visual analogue scale

*EORTC*-*QLQ*-*BR23* EORTC quality of life questionnaire breast cancer module

Regarding generic domains, all were highly correlated with their criterion measures with correlation coefficients in the range of 0.60 to 0.78. Finally, cancer-specific domains had a good correlation with values of 0.53 for appearance concerns and 0.60 in the distress over recurrence domain.

### Construct validity

**Fig. 1:**
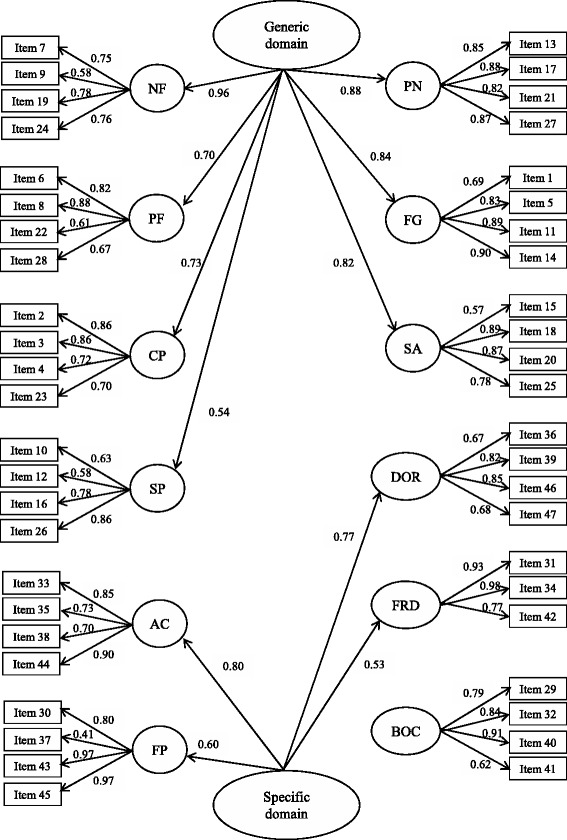
Second-order confirmatory factor analysis for categorical data of the QLACS questionnaire. Standardized parameters are shown. NF: Negative feelings; PF: Positive feelings; CP: Cognitive problems; SP: Sexual problems; PN: Pain; FG: Fatigue; SA: Social avoidance; AC: Appearance concerns; FP: Financial problems; DOR: Distress over recurrence; FRD: Family-related distress; BOC: Benefits of cancer. Fit indexes are as follows: RMSEA (90 % CI) = 0.061 (0.058 – 0.064); CFI = 0.929; TLI = 0.925

The results of the second-order CFA for the hypothesized model of 12 first-order factors and 2 s-order factors, the Generic and cancer-specific summary factors, provided satisfactory fit indices in all cases. The RMSEA value was 0.061, far below 0.08, and CFI and TLI values both exceeded 0.90 (0.929 and 0.925, respectively). All factor loadings were higher than 0.40 and statistically significant (*P* < 0.001) (Fig. 1).

Regarding the results of the Rasch analysis for the Generic scale, eight items showed misfit and were thus removed from the scale (Table 5). With the remaining 20 items, unidimensionality was supported with infit and outfit statistics of 0.7 to 1.3, except in item 8 with an outfit value slightly higher than 1.30. However, the PCA of the residuals did not yield additional factors with eigenvalues higher than 3, implying that the unidimensionality assumption was met. The item separation index was 7.70, higher than 2, indicating reliability higher than 0.80. The presence of DIF was not detected by type of cancer, gender or age group (data not shown). Correlation coefficients between residuals were all lower than 0.50, supporting local independence, and the functioning of the rating scale categories was adequate.

**Table 5:** Severity levels, standard errors, and goodness-of-fit indices of the QLACS Generic and cancer-specific scales, and the benefits of cancer and the sexual problems scales using Rasch analysis

Items	Item description	δ (logit)	SE	Infit MNSQ	Outfit MNSQ
Generic scale					
Item 1^a^	You had the energy to do the things you wanted to do	−0.16	0.03	1.23	1.28
Item 2	You had difficulty doing activities that require concentrating	0.38	0.04	0.77	0.84
Item 3	You were bothered by having a short attention span	0.27	0.04	1.20	1.12
Item 4	You had trouble remembering things	−0.04	0.03	1.16	1.22
Item 5	You felt fatigued	−0.19	0.03	0.83	0.85
Item 6	You felt happy	−0.40	0.03	1.25	1.33
Item 7	You felt blue or depressed	−0.09	0.03	0.87	0.94
Item 8	You enjoyed life	−0.38	0.03	1.30	1.32
Item 9	You worried about little things	−0.16	0.03	1.04	1.23
Item 10	You were bothered by being unable to function sexually	Removed			
Item 11	You didn’t have energy to do the things you wanted to do	−0.07	0.03	0.82	0.81
Item 12	You were dissatisfied with your sex life	Removed			
Item 13	You were bothered by pain that kept you from doing the things you wanted to do	−0.02	0.03	1.10	1.06
Item 14	You felt tired a lot	−0.33	0.03	0.71	0.72
Item 15	You were reluctant to start new relationships	Removed			
Item 16	You lacked interest in sex	Removed			
Item 17	Your mood was disrupted by pain or its treatment	0.07	0.04	1.05	0.99
Item 18	You avoided social gatherings	0.32	0.04	1.26	1.17
Item 19	You were bothered by mood swings	−0.15	0.03	0.80	0.86
Item 20	You avoided your friends	0.72	0.04	1.08	1.20
Item 21	You had aches or pains	−0.04	0.03	1.03	1.05
Item 22	You had a positive outlook on life	Removed			
Item 23	You were bothered by forgetting what you stated to do	0.24	0.04	0.89	1.10
Item 24	You felt anxious	−0.26	0.03	0.86	0.96
Item 25	You were reluctant to meet new people	Removed			
Item 26	You avoided sexual activity	Removed			
Item 27	Pain or its treatment interfered with your social activities	0.30	0.04	1.10	1.09
Item 28	You were content with your life	Removed			
Cancer-specific scale					
Item 30	You had financial problems because of the cost of cancer surgery or treatment	Removed			
Item 31	You worried that your family members were at risk of getting cancer	−0.46	0.04	1.04	1.01
Item 33	You were self-conscious about the way you look because of your cancer or its treatment	0.40	0.04	1.18	1.10
Item 34	You worried about whether your family members might have cancer-causing genes	−0.49	0.04	0.94	0.90
Item 35	You felt unattractive because of your cancer or its treatment	0.47	0.04	0.98	0.95
Item 36	You worried about dying from cancer	−0.14	0.04	1.02	1.02
Item 37	You had problems with insurance because of cancer	Removed			
Item 38	You were bothered by hair loss from cancer treatment	0.59	0.04	1.28	1.23
Item 39	You worried about cancer coming back	−0.55	0.04	0.81	0.83
Item 42	You worried about whether your family members should have genetic tests for cancer	−0.22	0.04	1.26	1.17
Item 43	You had money problems that arose because you had cancer	Removed			
Item 44	You felt people treated you differently because of changes to your appearance due to your cancer or its treatment	0.81	0.04	1.24	1.17
Item 45	You had financial problems due to a loss of income as a result of cancer	Removed			
Item 46	Whenever you felt a pain, you worried that it might be cancer again	−0.14	0.03	0.85	0.95
Item 47	You were preoccupied with concerns about cancer	−0.24	0.03	0.81	0.87
Benefits of cancer scale					
Item 29	You appreciated life more because of having had cancer	−0.38	0.03	1.15	1.15
Item 32	You realized that having had cancer helps you cope better with problems now	0.04	0.03	0.84	0.85
Item 40	You felt that cancer helped you to recognize what is important in life	−0.45	0.03	0.77	0.73
Item 41	You felt better able to deal with stress because of having had cancer	0.79	0.03	1.29	1.28
Sexual problems scale					
Item 10	You were bothered by being unable to function sexually	0.10	0.03	1.12	1.07
Item 12	You were dissatisfied with your sex life	0.01	0.03	0.94	0.89
Item 16	You lacked interest in sex	−0.09	0.03	0.98	0.90
Item 26	You avoided sexual activity	−0.02	0.03	0.98	0.89

^a^Reverse item

δ = level of severity (higher values indicate higher severity)

*SE* standard error, *MNSQ* mean square fit statistic

Item separation index of each model: 7.70 for the Generic scale, 11.89 for the cancer-specific scale, 14.27 for the benefits of cancer scale, and 2 for the sexual problems scale

As all sexual items misfit the Generic scale, we undertook a Rasch analysis separately for this domain (Table 5). The model showed unidimensionality, an item separation index of 2, no DIF by type of cancer, gender or age group (data not shown), and an adequate functioning of rating scale categories. However, local dependency was detected, with correlation coefficient between residual higher than 0.50 between the following pairs of items: item 10 and 12 with items 16 and 26.

The results of Rasch analysis for the cancer-specific scale (Table 5) showed four misfitting items, specifically items 30, 37, 43 and 45, all of them included in financial problems domain. After removing these four items, unidimensionality was supported with infit and outfit statistics between 0.7 and 1.3, and with no other factor besides the first one with eigenvalues higher than 3 in the PCA of the residuals. The item separation index was 11.89, higher than 2, correlation coefficients between residuals were all lower than 0.50, supporting local independence, and the functioning of the rating scale categories was adequate. However, the presence of DIF was detected in item 38 by type of cancer and gender (data not shown), being more difficult for patients with colorectal or prostate cancer, and for men, than for patients with breast cancer or women, and in item 44 being more difficult for patients with prostate cancer than for those with breast cancer.

Unidimensionality was supported in the Rasch analysis for the four items of the benefits of cancer scale (Table 5). The item separation index was 14.27, indicating good reliability. The presence of DIF was not detected by type of cancer, gender or age group (data not shown). Correlation coefficients between residuals were all lower than 0.50, supporting local independence, and the functioning of the rating scale categories was adequate.

**Fig. 2:**
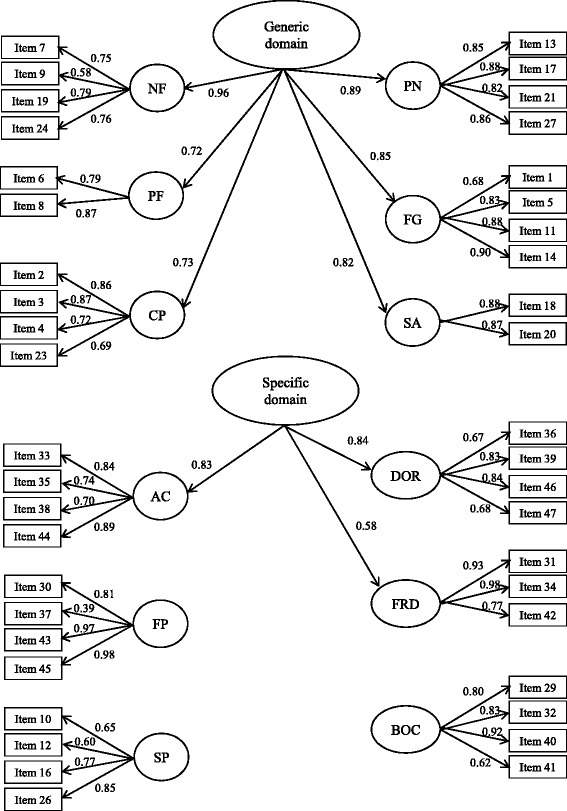
Second-order confirmatory factor analysis for categorical data of the QLACS questionnaire after removing four items and removing some relationships based on the results of Rasch analyses. Standardized parameters are shown. NF: Negative feelings; PF: Positive feelings; CP: Cognitive problems; SP: Sexual problems; PN: Pain; FG: Fatigue; SA: Social avoidance; AC: Appearance concerns; FP: Financial problems; DOR: Distress over recurrence; FRD: Family-related distress; BOC: Benefits of cancer. Fit indexes are as follows: RMSEA (90 % CI) = 0.061 (0.057 – 0.064); CFI = 0.939; TLI = 0.935

Based on the results of Rasch analyses, we propose a model in which four items were deleted (items 22 and 28 from PF and items 15 and 25 from SA), and the second-order generic factor would not affect the SP first-order factor, and the second-order specific factor would not affect the FP first-order factor. The results of the second-order CFA for this model (Fig. 2) provided satisfactory fit indices and almost identical to those obtained for the original model (Fig. 1). The RMSEA was 0.061, and the CFI and TLI values were 0.939 and 0.935, respectively.

## Discussion

This study provides data from the classical psychometric and Rasch approach on the validation of the Spanish version of the QLACS questionnaire. Although this questionnaire was developed for long-term cancer survivors, the findings of this study showed that QLACS is a valid and reliable instrument to be used in short-term cancer survivors in Spanish population.

In the year 2008 [28], the need for psychometrically credible quality of life instruments for cancer survivors who were in 1–5 years post diagnosis phase had already been detected. Although the QLACS questionnaire was developed originally for long-term survivors, it has recently demonstrated its usefulness in shorter-term cancer survivors in its original language [8, 9]. As a consequence, this study has tried to provide some information about the reliability and validity of the QLACS questionnaire in another language.

In terms of internal consistency, our Cronbach’s alphas are in the range of 0.73–0.86 for all domains and summary scales. They are similar to values found in the original instrument that were between 0.72 and 0.91 [5] and a bit lower than those reported by Ashley et al. [8] that were between 0.75 and 0.95, in a sample of similar characteristics of short-term cancer survivors and those reported by Sohl et al. [9] which were in the 0.79–0.91 range, in short-term breast cancer survivors. Likewise, all coefficients for item-domain and item-scale correlations have shown adequate values and are higher than the accepted values with the only exception of item 37. On the other hand, test-retest reliability, measured in stable patients, can be considered as appropriate in all domains [17]. These facts seem to assure the reliability of the instrument.

Although both summary scales are free from ceiling or floor effects, our data show a floor effect (minimal score) in four domains. Three of them: social avoidance, appearance concerns and financial problems showed the same floor effect as in a previous validation work [8] with the same cancer diagnosis carried out as well with short-term survivors. In addition, in two of them: social avoidance and financial problems, a recent work [9], has found the same floor effect. In a sample of breast cancer survivors [29], and in her original work [5], there also were floor effects in appearance concerns and financial problems. One possible explanation for the effect in social avoidance and appearance concerns domains could be that cancer locations included in the study are not the more problematic regarding physical problems 2–3 years post treatment. Possibly the acute appearance problems which can influence social avoidance due to the treatment effects have already been resolved in this stage of the disease in these cancer locations. The floor effect in financial problems possibly could be explained because questions included in the questionnaire are closely related to the cost of the disease, its treatment and insurance plans, and in Spain these issues were, in general, covered by the National Health Service. The fourth dimension is pain, which shows values close to the original work [5], the work of previous validation in shorter-term survivors [8], another carried out with breast cancer survivors [29] and with short-term breast cancer survivors [9]. However, there was only one dimension with ceiling effect, family-related distress. One possible explanation might be that the patients are increasingly aware of the influence of genetics on the development of some types of cancers.

Regarding feasibility, there were several items with missing values above 5 %. As in the study of Ashley [8], the four items of sexual problems domain, as well as items 28 (positive feelings), 27 (pain) and 15 (social avoidance presented missing data. It is well known that the presence of missing data in sexual aspects is a frequent finding in the research field. Regarding item 15, we agree with the explanation given by Ashley et al. [8] about the possible interpretation of the meaning of the item as sexual instead of social relationships. We do not have a satisfactory explanation for items 27 and 28.

The questionnaire seems to have good convergent validity in the studied domains. As in the original work, Generic domains have higher correlation coefficients than Specific domains.

Our second-order CFA results indicate that the structure of the questionnaire does have adequate structural validity. Considering the results of Rasch analysis for the Generic scale, we found eight misfitting items: the four items of sexual problems domain (items 10, 12, 16 and 26), two items of the positive feeling domain (items 22 and 28), and two items of the social avoidance domain (items 15 and 25). Ashley et al. [8], in the only study in which a Rasch analysis is applied to the questionnaire, also find the four items of the sexual problems scale and item 15 of the social avoidance domain to be problematic. Regarding items 22 and 28 of the positive feeling domain, the CFA results showed factor loadings for these two items much lower than those of the other two items in the same domain. Considering the results of the Rasch analysis for the sexual problems domain separately, we found local dependency. Our results, suggested the existence of two subscales, one of function (items 10 and 12) and another of interest (items 16 and 26). These results agree with the results obtained by Ashley et al. [8], and also with the results obtained by the original authors [5], since in the exploratory factor analysis they presented these four items in two separate factors.

Regarding the results of Rasch analysis for the cancer-specific scale, we found the four items of the financial problems scale to be misfit. This result does not agree with those obtained by Ashley et al. [8]. Furthermore, DIF was found for item 38 between patients with colorectal or prostate cancer with respect to those with breast cancer, and between men and women. DIF was also found in item 44 between patients with prostate cancer and those with breast cancer.

Like Ashley et al. [8], we also found that all items of the benefits of cancer scale showed fit to the Rasch model, demonstrated unidimensionality, adequate item separation index, and no DIF by gender, type of cancer or age group.

This study has some limitations; it is centered on patients with breast, prostate or colorectal cancer, so the results may not extrapolate to other cancer locations. This study has been carried out with non-metastatic patients and without local-recurrence. Finally, we have been unable to study convergent validity in all domains due to the lack of adequate dimensions in other used instruments.

## Conclusions

The Spanish version of the Quality of Life in Adult Cancer Survivors (QLACS) has shown good reliability and validity and the findings of this study support its use to assess quality of life in short-term cancer survivors. As a consequence, the questionnaire may be used to establish quality of life in Spanish short-term survivors from breast, prostate or colorectal cancer.

## Additional file

Additional file 1:
**The Spanish version of the Quality of Life in Adults Cancer Survivors (QLACS) questionnaire.** (PDF 58 kb)
