# Supplementary material for: Cross-cultural adaptation, reliability and validity of the Spanish version of the Quality of Life in Adult Cancer Survivors (QLACS) questionnaire: application in a sample of short-term survivors
Source: Health Qual Life Outcomes. 2015 Nov 16;13:182. doi: 10.1186/s12955-015-0378-2 (PMC4647305; doi:10.1186/s12955-015-0378-2)
Supplement: Additional file 1: — The Spanish version of the Quality of Life in Adults Cancer Survivors (QLACS) questionnaire. (PDF 58 kb) [file 12955_2015_378_MOESM1_ESM.pdf]

**The Spanish version of the Quality of Life in Adults Cancer Survivors (QLACS) questionnaire**

Instrucciones: Nos gustaría preguntarte sobre algunos aspectos que pueden afectar a la calidad de vida de las personas. Algunas de estas afirmaciones pueden parecer similares, pero por favor asegúrate de responder a todas. A continuación hay una escala que va del "nunca" al "siempre". Por favor, indica con qué frecuencia has experimentado las siguientes afirmaciones en las últimas cuatro semanas. [RODEA CON UN CÍRCULO SOLAMENTE UNA RESPUESTA POR PREGUNTA]

| | Nunca | Casi nunca | A veces | Habitualmente | Con frecuencia | Muy a menudo | Siempre |
|---|---|---|---|---|---|---|---|
| **En las últimas 4 semanas…** | | | | | | | |
| 1. Has tenido energía para hacer las cosas que querías hacer. | 1 | 2 | 3 | 4 | 5 | 6 | 7 |
| 2. Has tenido dificultad para hacer actividades que necesitan concentración. | 1 | 2 | 3 | 4 | 5 | 6 | 7 |
| 3. Te ha molestado no poder mantener la atención durante un período prolongado de tiempo. | 1 | 2 | 3 | 4 | 5 | 6 | 7 |
| 4. Has tenido dificultad para recordar las cosas. | 1 | 2 | 3 | 4 | 5 | 6 | 7 |
| 5. Te has sentido fatigado. | 1 | 2 | 3 | 4 | 5 | 6 | 7 |
| 6. Te has sentido feliz. | 1 | 2 | 3 | 4 | 5 | 6 | 7 |
| 7. Te has sentido triste o deprimido. | 1 | 2 | 3 | 4 | 5 | 6 | 7 |
| 8. Has disfrutado de la vida. | 1 | 2 | 3 | 4 | 5 | 6 | 7 |
| 9. Te has preocupado por cosas insignificantes. | 1 | 2 | 3 | 4 | 5 | 6 | 7 |
| 10. Te ha molestado ser incapaz de mantener relaciones sexuales. | 1 | 2 | 3 | 4 | 5 | 6 | 7 |
| 11. Te ha faltado energía para hacer las cosas que querías hacer. | 1 | 2 | 3 | 4 | 5 | 6 | 7 |
| 12. Te has sentido insatisfecho con tu vida sexual. | 1 | 2 | 3 | 4 | 5 | 6 | 7 |
| 13. Te ha molestado el dolor que te ha impedido hacer las cosas que querías hacer. | 1 | 2 | 3 | 4 | 5 | 6 | 7 |
| 14. Te has sentido cansado a menudo. | 1 | 2 | 3 | 4 | 5 | 6 | 7 |
| 15. Te has mostrado reacio a comenzar nuevas relaciones. | 1 | 2 | 3 | 4 | 5 | 6 | 7 |
| 16. Te ha faltado interés por el sexo. | 1 | 2 | 3 | 4 | 5 | 6 | 7 |
| 17. Tu humor se ha visto afectado por el dolor o su tratamiento. | 1 | 2 | 3 | 4 | 5 | 6 | 7 |
| 18. Has evitado las reuniones sociales. | 1 | 2 | 3 | 4 | 5 | 6 | 7 |
| 19. Has experimentado cambios de humor. | 1 | 2 | 3 | 4 | 5 | 6 | 7 |
| 20. Has evitado a tus amigos. | 1 | 2 | 3 | 4 | 5 | 6 | 7 |
| 21. Has tenido dolor. | 1 | 2 | 3 | 4 | 5 | 6 | 7 |
| 22. Has tenido una actitud positiva ante la vida. | 1 | 2 | 3 | 4 | 5 | 6 | 7 |
| 23. Te ha molestado olvidar lo que habías empezado a hacer. | 1 | 2 | 3 | 4 | 5 | 6 | 7 |
| 24. Te has sentido inquieto. | 1 | 2 | 3 | 4 | 5 | 6 | 7 |
| 25. Te has mostrado reacio a conocer a nuevas personas. | 1 | 2 | 3 | 4 | 5 | 6 | 7 |
| 26. Has evitado la actividad sexual. | 1 | 2 | 3 | 4 | 5 | 6 | 7 |
| 27. El dolor o su tratamiento han afectado a tus actividades sociales. | 1 | 2 | 3 | 4 | 5 | 6 | 7 |

| Las siguientes afirmaciones se refieren específicamente a los efectos del cáncer o su tratamiento. De nuevo, por cada frase, indica con qué frecuencia has experimentado estas afirmaciones en las últimas cuatro semanas. | | | | | | | |
|---|---|---|---|---|---|---|---|
| | Nunca | Casi nunca | A veces | Habitualmente | Con frecuencia | Muy a menudo | Siempre |
| 28. Te has sentido contento con tu vida. | 1 | 2 | 3 | 4 | 5 | 6 | 7 |
| 29. Has valorado más la vida por haber tenido cáncer. | 1 | 2 | 3 | 4 | 5 | 6 | 7 |
| 30. Has tenido problemas económicos debido al coste de la intervención quirúrgica o tratamiento para el cáncer. | 1 | 2 | 3 | 4 | 5 | 6 | 7 |
| 31. Te ha preocupado que tus familiares tuvieran riesgo de padecer cáncer. | 1 | 2 | 3 | 4 | 5 | 6 | 7 |
| 32. Te has dado cuenta de que haber tenido cáncer te ayuda ahora a sobrellevar mejor los problemas. | 1 | 2 | 3 | 4 | 5 | 6 | 7 |
| 33. Te ha preocupado tu aspecto físico debido al cáncer o su tratamiento. | 1 | 2 | 3 | 4 | 5 | 6 | 7 |
| 34. Te ha preocupado el que tus familiares pudieran tener genes relacionados con el cáncer. | 1 | 2 | 3 | 4 | 5 | 6 | 7 |
| 35. No te sentías atractivo por el cáncer o su tratamiento. | 1 | 2 | 3 | 4 | 5 | 6 | 7 |
| 36. Te ha preocupado poder morir de cáncer. | 1 | 2 | 3 | 4 | 5 | 6 | 7 |
| 37. Has tenido problemas con el seguro debido al cáncer. | 1 | 2 | 3 | 4 | 5 | 6 | 7 |
| 38. Te ha molestado la pérdida de cabello a causa del tratamiento del cáncer. | 1 | 2 | 3 | 4 | 5 | 6 | 7 |
| 39. Te ha preocupado que el cáncer recurriera. | 1 | 2 | 3 | 4 | 5 | 6 | 7 |
| 40. Has sentido que el cáncer te ha ayudado a reconocer lo que es verdaderamente importante en la vida. | 1 | 2 | 3 | 4 | 5 | 6 | 7 |
| 41. Has sentido que podías sobrellevar mejor el estrés por haber tenido cáncer. | 1 | 2 | 3 | 4 | 5 | 6 | 7 |
| 42. Te ha preocupado el hecho de si los miembros de tu familia debían someterse a pruebas genéticas para detectar el cáncer. | 1 | 2 | 3 | 4 | 5 | 6 | 7 |
| 43. Has tenido problemas monetarios a causa del cáncer. | 1 | 2 | 3 | 4 | 5 | 6 | 7 |
| 44. Has sentido que la gente te trataba de forma diferente por los cambios en tu aspecto físico debidos al cáncer o su tratamiento. | 1 | 2 | 3 | 4 | 5 | 6 | 7 |
| 45. Has tenido problemas económicos por la pérdida de ingresos como resultado del cáncer. | 1 | 2 | 3 | 4 | 5 | 6 | 7 |
| 46. Cada vez que has sentido dolor, te preocupaba que fuera de nuevo por el cáncer. | 1 | 2 | 3 | 4 | 5 | 6 | 7 |
| 47. Te has preocupado con cuestiones relacionadas con el cáncer. | 1 | 2 | 3 | 4 | 5 | 6 | 7 |
